# Stress Resilience of Spermatozoa and Blood Mononuclear Cells without Prion Protein

**DOI:** 10.3389/fmolb.2018.00001

**Published:** 2018-01-24

**Authors:** Malin R. Reiten, Giulia Malachin, Elisabeth Kommisrud, Gunn C. Østby, Karin E. Waterhouse, Anette K. Krogenæs, Anna Kusnierczyk, Magnar Bjørås, Clara M. O. Jalland, Liv Heidi Nekså, Susan S. Røed, Else-Berit Stenseth, Frøydis D. Myromslien, Teklu T. Zeremichael, Maren K. Bakkebø, Arild Espenes, Michael A. Tranulis

**Affiliations:** ^1^Faculty of Veterinary Medicine and Biosciences, Norwegian University of Life Sciences, Oslo, Norway; ^2^Faculty of Education and Natural Sciences, Inland University of Applied Sciences, Hamar, Norway; ^3^Spermvital AS Holsetgata, Hamar, Norway; ^4^Department of Cancer Research and Molecular Medicine, Norwegian University of Science and Technology, Trondheim, Norway

**Keywords:** prion protein, stress resilience, spermatocytes, peripheral blood mononuclear cells, goat model, testes, ROS stress

## Abstract

The cellular prion protein PrP^C^ is highly expressed in neurons, but also present in non-neuronal tissues, including the testicles and spermatozoa. Most immune cells and their bone marrow precursors also express PrP^C^. Clearly, this protein operates in highly diverse cellular contexts. Investigations into putative stress-protective roles for PrP^C^ have resulted in an array of functions, such as inhibition of apoptosis, stimulation of anti-oxidant enzymes, scavenging roles, and a role in nuclear DNA repair. We have studied stress resilience of spermatozoa and peripheral blood mononuclear cells (PBMCs) derived from non-transgenic goats that lack PrP^C^ (*PRNP*^Ter/Ter^) compared with cells from normal (*PRNP*^+/+^) goats. Spermatozoa were analyzed for freeze tolerance, DNA integrity, viability, motility, ATP levels, and acrosome intactness at rest and after acute stress, induced by Cu^2+^ ions, as well as levels of reactive oxygen species (ROS) after exposure to FeSO_4_ and H_2_O_2_. Surprisingly, PrP^C^-negative spermatozoa reacted similarly to normal spermatozoa in all read-outs. Moreover, *in vitro* exposure of PBMCs to Doxorubicin, H_2_O_2_ and methyl methanesulfonate (MMS), revealed no effect of PrP^C^ on cellular survival or global accumulation of DNA damage. Similar results were obtained with human neuroblastoma (SH-SY5Y) cell lines stably expressing varying levels of PrP^C^. RNA sequencing of PBMCs (*n* = 8 of *PRNP*^+/+^ and *PRNP*^Ter/Ter^) showed that basal level expression of genes encoding DNA repair enzymes, ROS scavenging, and antioxidant enzymes were unaffected by the absence of PrP^C^. Data presented here questions the *in vitro* cytoprotective roles previously attributed to PrP^C^, although not excluding such functions in other cell types or tissues during inflammatory stress.

## Introduction

The cellular prion protein (PrP^C^) is the substrate for prion propagation in which the protein is misfolded to the pathogenic scrapie conformer (PrP^Sc^) (Prusiner, 1993). Neurons have limited capacity to degrade or otherwise dispose safely of PrP^Sc^, which ultimately causes their demise. Aggregates of PrP^Sc^, containing infectious prions, in the central nervous system (CNS) and to varying degrees in peripheral organs, are pathognomonic for incurable prion diseases such as Creutzfeldt Jakob disease in humans, scrapie in sheep, and chronic wasting disease in deer (Aguzzi and Calella, 2009). Understanding the physiological function of PrP^C^ is important for deciphering the pathogenesis of prion diseases and for development of prevention strategies.

During its synthesis, PrP^C^ is translocated into the endoplasmic reticulum and the secretory pathway. It undergoes several post-translational modifications, including attachment of two N-glycans and a C-terminal glycosylphosphatidylinositol (GPI) anchor that ultimately tethers the protein to the outer leaflet of the plasma membrane (Stahl et al., 1987). Many aspects concerning PrP^C^'s sub-cellular localization, post-translational modifications, and participation in various cellular processes are still incompletely understood.

The protein is abundantly present in the central and peripheral nervous system, but also, at lower levels, in most other tissues. The widespread expression of the gene encoding PrP^C^ (*Prnp*) already during embryonal development (Manson et al., 1992; Tremblay et al., 2007) and in adult animals (Bendheim et al., 1992), suggests that it functions in diverse physiological and cellular contexts. Initial analysis of mice with genetic knockout of PrP^C^ (*Prnp*^−/−^) showed that they developed and remained healthy without displaying any aberrant phenotypes (Bueler et al., 1992; Manson et al., 1994), apart from being completely resistant to prion disease (Büeler et al., 1993). Subsequently, a large catalog of putative PrP^C^ functions has evolved, including that PrP^C^ is cytoprotective (Mitteregger et al., 2007). Experiments involving hypoxic brain damage (McLennan et al., 2004; Weise et al., 2004, 2006; Shyu et al., 2005; Spudich et al., 2005) or severe inflammation, such as experimental autoimmune encephalomyelitis (Tsutsui et al., 2008; Gourdain et al., 2012) or experimentally induced colitis (Martin et al., 2011) showed that pathologies were exacerbated in animals without PrP^C^ expression.

By exposing cells to various forms of stress *in vitro*, it has been demonstrated that PrP^C^ contributes to cellular protection by modulating different pathways. For instance, through inhibition of Bax-mediated apoptosis (Kuwahara et al., 1999; Bounhar et al., 2001; Roucou et al., 2005), or by stimulation of pro-survival signaling (Chiarini et al., 2002; Lopes et al., 2005) through cell-surface interaction with the extracellular co-chaperone Sti1. It has also been shown that PrP^C^ can contribute to increased antioxidant defense (Brown et al., 2002; Rachidi et al., 2003; Haigh et al., 2015) and upon translocation to the cell nucleus to augmented AP endonuclease 1-driven DNA repair (Bravard et al., 2015). There are also examples in the literature of PrP^C^ conferring variable (Yu et al., 2012) and even reduced (Paitel et al., 2003) viability under certain conditions of stress. Despite the perplexing pleiotropy in PrP^C^ functions, several lines of evidence, derived from different experimental modalities, converge in highlighting the importance of the Cu^2+^-binding N-terminal domain of PrP^C^ for its protective properties (Dupiereux et al., 2008; Guillot-Sestier et al., 2009; Haigh et al., 2015). Importantly, this part of PrP^C^ can be liberated through proteolytic cleavage in response to oxidative stress (McMahon et al., 2001; Watt et al., 2005).

PrP^C^ is present at relatively high levels in the testicles, epididymis, and seminal fluid, and at lower levels, on the surface of ejaculated spermatozoa (Shaked et al., 1999; Ford et al., 2002; Peoc'h et al., 2002; Fujisawa et al., 2004). It has been observed that spermatozoa derived from *Prnp*^−/−^ mice are highly susceptible to Cu^2+^-induced oxidative stress compared with wild-type mice (Shaked et al., 1999), suggesting that PrP^C^ by virtue of its Cu^2+^-binding properties contributes significantly to the protection of spermatozoa against reactive oxygen species (ROS) stress; however, not critical for fertility.

Taken together, the mechanisms of PrP^C^-mediated stress protection are incompletely understood and previously assigned stress-protective roles of PrP^C^ have recently been questioned (reviewed in Castle and Gill, 2017), pointing to the need for reassessment and cross-validation by newly developed animal models. In the present investigation, we addressed this by examining oxidative and genotoxic stress resilience of ejaculated spermatozoa and circulating mononuclear cells derived from a naturally occurring line of goats that completely lack PrP^C^ (*PRNP*^Ter/Ter^) in comparison with wild-type goats of the same breed (Benestad et al., 2012). Animals carrying the *PRNP*^*Ter*^ allele do not display aberrant behavior, such as anxiety, or other clinically recognizable phenotypes. However, detailed analysis at resting state (Reiten et al., 2015; Malachin et al., 2017) and under inflammatory stress induced by lipopolysaccharide (LPS) (Salvesen et al., 2017) have provided data suggesting that PrP^C^ has a modulatory role in certain immunological pathways, such as type I interferon signaling.

## Materials and methods

### Animals and sample material

Age-and gender-matched goats of the Norwegian Dairy Goat breed born between February–March 2016, and genotyped as either normal (*PRNP*^+/+^) or PrP^C^ deficient (*PRNP*^Ter/Ter^), were included in the study. Animals were held in a farmhouse environment and showed no signs of abnormal health issues throughout the sampling period. The study was approved by the Committee on the Ethics of Animal Experiments by The Norwegian Animal Research Authority (ID No. 8058).

### Semen collection and cryopreservation

Two groups of bucks, *PRNP*^+/+^ (*n* = 4) and *PRNP*^Ter/Ter^ (*n* = 4) genotypes, with mean age 208 and 223 days, respectively, were used. The bucks were housed at the Norwegian sheep and goat breeders AI station at Hjermstad (Norway), and allowed an acclimatization period of 2 weeks. Following a training period, semen samples were successfully collected using an artificial vagina while the bucks were mounting an estrous goat.

The volume of the ejaculates was registered, after which the spermatozoa concentration was quickly assessed by spectrophotometer in order to determine the correct dilution factor to attain a standardized concentration of 800 × 10^6^ spermatozoa/ml. The ejaculates were kept at 35°C for 10 min, before dilution to a final volume of 15 ml using AndroMed® (Minitübe, Tiefenbach, Germany) extender. After 15 min at room temperature, the ejaculates were placed in a water bath at 5°C and kept at this temperature for 2 h, prior to centrifugation at 800 × g for 10 min. Some of the supernatant was carefully removed leaving the final pre-calculated volume. Spermatozoa were re-suspended by gentle mixing before filling into 0.25 ml French mini straws (IMV, L'Aigle, France). The straws were placed on ramps and cryopreserved by a cooling rate of 2°C/min from +5° to −10°C and from −10° to −150°C with cooling rate of 40°C/min, and thereafter plunged into liquid nitrogen (LN2). The straws were put in goblets and stored in LN2. When semen collection was finalized, the bucks were euthanized by an intravenous injection of pentobarbital (Euthasol vet, Richter Pharma, Austria) and tissue samples were immediately collected and treated as specified for subsequent storage and analysis.

### Immunohistochemistry and immunofluorescence of testicle and epididymis

For PrP^C^ detection in the testicle and epididymis, tissues from one buck of each genotype were used. Tissues were snap frozen in liquid nitrogen and stored at −80°C. Cryosections (12 μm) were taken of frozen tissue samples and the slides allowed to dry before further use. Tissue sections were fixed in formolcalcium prior to antibody labeling. Washing with PBS followed after each step. *PRNP*^Ter/Ter^ tissue functioned as negative control.

For immunohistochemistry (IHC), an Envision anti-mouse kit (Dako, Agilent, Santa Clara, CA) was used for endogenous peroxidase blocking following the manufacturer's procedures. Goat serum was added for Fc blocking prior to incubation with primary antibodies for 45 min. For IHC, anti-prion antibodies 6H4 (mouse IgG1k, Prionics, ThermoFisher Scientific, Waltham, MA) and SAF32 (mouse IgG2b, SpiBio, Bertin pharma, Montigny le Bretonneux, France) were used, while for immunofluorescence (IF), 6H4 only was used. Secondary anti-mouse antibodies from the kit were added for 45 min, and stained with 3,3′-Diaminobenzidine (DAB), before mounting with aqueous medium. All slides were evaluated by standard light microscopy and photos were taken with a Leica EC3 camera (Leica Camera AG, Wetzlar, Germany).

For IF, goat serum was added for Fc blocking prior to incubation with primary antibodies for 3 h. 6H4 (mouse IgG1k) was used to detect PrP^C^ and c-kit/CD117 (rabbit polyclonal, Dako, Agilent) was used to detect germ cells. Secondary antibodies for PrP^C^ (Alexa 488 IgG1 goat anti-mouse, Molecular Probes, ThermoFisher Scientific) and c-kit (Alexa 594 IgG (H+L) goat anti-rabbit, Molecular Probes, ThermoFisher Scientific) were allowed to incubate for 1 h. ProLong™ Diamond Antifade Mountant with DAPI (ThermoFisher Scientific) was used for mounting and nuclei staining. Fluorescence was visualized with an Axio Imager 2 Research Microscope (Zeiss, Oberkochen, Germany) and images were processed in Zen (Zeiss).

### Histochemistry for lipids with oil red O

Four bucks of each genotype were investigated. Cryosections from the testicle were fixed by a mixture of 40% formaldehyde and 70% ethanol 1:9 v/v for 5 min and stained by a standard protocol [Oil red O (ORO), Schmid GMBH & Co]. Slides were counterstained with hematoxylin and mounted with aqueous medium before evaluation by standard light microscopy.

### Western blot

To remove seminal plasma prior to analysis, spermatozoa from one straw of each genotype were washed twice in 1 ml of PBS by centrifugation at 800 rpm for 5 min, followed by careful removal of the supernatant. Washed spermatozoa and testicle tissue samples were lysed in homogenizer buffer (Tris HCl 50 μM, NaCl 150 mM, EDTA 1 mM, DOC 0.25%, NP40 1%) supplemented with protease inhibitor cocktail 2x (Complete, Roche, Merck Life Science, Darmstadt, Germany). Twenty micrograms of protein, measured by the Protein assay (Bio-Rad, Hercules, CA), were deglycosylated with PNGase-F according to the manufacturer's instructions (New England Biolabs, Ipswich, MA). Twenty micrograms of protein and the deglycosylated samples were mixed with SDS Loading buffer and Sample Reducing agent (Invitrogen, ThermoFisher Scientific), heated for 10 min at 95°C before separation by sodium dodecyl sulfate polyacrylamide gel electrophoresis (12% Criterion gel, Bio-Rad), and transferred to a polyvinylidene difluoride (PVDF) membrane (GE Healthcare, Chicago, Il). Membranes were blocked in 5% milk TBST, and probed with primary antibodies; P4 mouse anti-PrP^C^ antibody (Ridascreen Biopharm, Darmstadt, Germany) 1:100, anti-c-kit/CD117 (rabbit polyclonal, Dako, Agilent) 1:500 and anti-beta Actin (Mouse monoclonal, ThermoFisher Scientific, Waltham, MA, USA) 1:20,000. Secondary anti-mouse or anti-rabbit antibodies conjugated with Alkaline Phosphatase (AP) were used for the detection, developed with Enhanced chemiluminescence (ECF) reagent (GE Healthcare) and Typhoon 9200 (Amersham Bioscience, GE Healthcare).

### CuCl_2_ treatment of spermatozoa

Two straws from each buck were thawed in a water bath at 35°C for 30 s. The pooled straws were aliquoted, and CuCl_2_ was added and gently mixed. Three concentrations of CuCl_2_ were used (100, 150, or 200 μg/ml), while no addition served as control. A stock solution of 1 mg/ml CuCl_2_ in PBS was used in combination with pure PBS to obtain the two concentrations. Semen samples were analyzed after 0, 30, 60, 90, and 120 min incubation at 35°C.

### Plasma membrane and acrosome integrity analysis of spermatozoa

Spermatozoa plasma membrane integrity (spermatozoa viability) was assessed using Propidium iodide (PI, L-7011, LIVE/DEAD®Sperm Viability Kit, Molecular Probes, ThermoFisher Scientific) to discriminate between live and dead (PI positive) spermatozoa. The proportion of acrosome reacted/degenerated spermatozoa was identified using the peanut (*Arachis hypogaea*) agglutinin (PNA) lectin conjugated with Alexa Fluor® 488 (PNA-Alexa 488, L21409, Invitrogen, ThermoFisher Scientific). Prior to flow cytometry analysis, the spermatozoa were stained for 10 min at room temperature in a PBS staining solution with a final concentration of ≈1.5 × 10^6^ spermatozoa/ml, 0.47 μM PI and 49 ng/ml PNA. Four replicates of each semen sample were analyzed. The reliability of the PI staining was confirmed in control samples double stained with both PI and SYBR-14 (Molecular Probes, ThermoFisher Scientific). Upon staining, analysis of the spermatozoa was performed using a Cell Lab Quanta TM SC MPL flow cytometer (Beckman Coulter, Fullerton, USA). The instrument was equipped with a 22 mW argon laser with excitation at 488 nm. Data was analyzed using Cell Lab Quanta SC MPL Analysis software program (Beckman Coulter). To identify the spermatozoa, a combination of electronic volume (EV) and side scatter (SS) signals were used, as described by Standerholen et al. (2014). Fluorescence detection and gating of the acrosome intact (AI) and acrosome intact live (AIL) spermatozoa was also performed according to Standerholen et al. (2014).

### Spermatozoa motility analysis by CASA

Spermatozoa motility analysis was performed using Sperm Class Analyzer (SCA Evolution, version 6.1; Microptic S.L., Barcelona, Spain) CASA system. Three microliters of each sample was loaded into a pre-warmed (37°C) standardized Leja 4-chamber microscope slide (Leja products, Nieuw-Vennep, The Netherlands) and analyzed using a phase contrast microscope (Nikon Eclipse, Nicon Group, Japan) equipped with Basler digital camera (Basler Vision Technologies, Basler AG, Ahrensburg, Germany). For each semen sample (*n* = 4), two replicates were analyzed, and for each replicate, eight microscopic fields were scanned, with a total of at least 500 cells per sample, and mean of the eight fields was presented. The motility parameters analyzed were total motility and progressive motility. The instrument settings for the analysis were; spermatozoa head area between 25 and 75 μm^2^; frame rate of 25 frames/s; immotile spermatozoa defined with an average path velocity below 10 μm/s.

### Assessment of ATP content

The ATP content was determined using the CellTiter-Glo® Luminescent Cell Viability Assay (Promega, Madison, WI). This method was previously adopted for the evaluation of the ATP content in boar semen (Long and Guthrie, 2006); however, the optimal spermatozoa number for analysis of goat semen was determined in the present study. For preparation of ATP standard curve samples, ATP disodium salt hydrate (A7699-1G, Sigma-Aldrich, Merck Life Science) was prepared in PBS to obtain the following ATP concentrations: 0, 40, 80, 200, 800, and 1,000 nM. Prior to analysis, goat semen was diluted to 1.5 × 10^6^ spermatozoa/ml in PBS, and 50 μl samples transferred to wells in a white 96-well microtiter plate (NUNC™, ThermoFisher Scientific). Subsequently, 50 μl CellTiter-Glo® Reagent was added to each well and the mixture was gently shaken for 2 min in a rotary shaker to induce cell lysis. After further incubation for 15 min at room temperature, bioluminescence measurement was performed using a FLUOstar OPTIMA multiwell plate reader (BMG LABTECH GmbH, Offenburg, Germany) with MARS data analysis software (Version 1.10, BMG LABTECH GmbH). Software setting was luminescence mode with gain 2900 and measurement time interval 0.5 s. By use of the ATP standard curve, the bioluminescence value for each sample, measured in relative luminescence units (RLU), was converted to the corresponding ATP-value in nM. An average of three replicates was used for statistical analysis.

### ROS analysis of spermatozoa

One semen straw from each buck was thawed for 8 s in a 70°C water bath. The spermatozoa concentration was measured and the semen was diluted in PBS prior to staining with fluorescence markers to a final concentration of 5 × 10^6^ spermatozoa/ml. Hoechst 34580 (1.25 μM, Invitrogen, ThermoFisher Scientific) and Mitotracker Orange CMTMRos (MO, 0.15 μM, Invitrogen, ThermoFisher Scientific) were used to eliminate non-spermatozoa events based on DNA and mitochondrial staining, respectively. Propidium Iodide (PI, 5 μg/ml, Invitrogen, ThermoFisher Scientific) was used to discriminate between plasma membrane-intact and degenerated spermatozoa, while CellROX®Deep Red Reagent (CRR, 5 μM, Invitrogen, ThermoFisher Scientific) was used to assess levels of ROS as a measure of cellular oxidative stress.

Basal levels of ROS as well as levels after induction of oxidative stress was assessed, both in duplicate samples. Semen samples were subjected to oxidative stress with 500 μM FeSO_4_·7H_2_O and 196 μM H_2_O_2_ to induce the Fenton reaction. At the same time point, the CRR and the Hoechst were added and the samples were incubated for 15 min at room temperature, after which, the markers MO and PI were added and the samples were incubated for another 15 min.

The semen samples were analyzed on a Navios flow cytometer (Beckman Coulter) equipped with a 488 nM (blue), a 638 nM (red), and a 405 nM (violet) diode laser. The two markers MO and PI were exited using the blue laser and fluorescence emission was collected using a 560–590 BP filter (FL2) and a 680–700 nM BP filter (FL4), respectively. Hoechst 34580 was exited using the blue laser and CRR using the red laser, while fluorescence emission was detected using a 530–570 nM BP filter (FL10) and a 650–670 nM BP (FL6), respectively. The instrument was checked daily for optical alignment by running Flow-Check beads (6605359, Beckman Coulter). An unstained semen sample was included as negative fluorescence control. Compensation was performed prior to collection of data with unstained semen samples and samples stained singularly with each fluorescence marker.

The flow cytometry-generated data were analyzed in FCS express software analysis program (De Novo Software, Canada). Computer-defined gates were set in a cytogram of Hoechst vs. MO to identify the spermatozoa (Hoechst and MO positive). Spermatozoa viability, defined as spermatozoa with a functional mitochondrial staining (MO-positive) and intact plasma membrane (PI negative), was estimated by use of an MO vs. PI cytogram. A cytogram of CRR vs. PI was used to determine the proportions of spermatozoa with different levels of ROS within the viable spermatozoa population (i.e., non-viable spermatozoa were excluded, **Figure 4**).

### Collection of PBMCs

Blood samples from animals of both genotypes were collected from the jugular vein into EDTA tubes and kept at room temperature until analysis. Peripheral blood mononuclear cells (PBMCs) were harvested and cultured as previously described (Reiten et al., 2015). For the PBMC experiments, cells from individual animals were used as biological replicates. A total of four animals of each genotype were included in the experiments with oxidative stressors, and the experiment was repeated once with four of the same animals, two of each genotype.

### SH-SY5Y cell culture

Human neuroblastoma SH-SY5Y cells (Sigma-Aldrich, Merck Life Science) were cultured and transfected with a plasmid construct encoding human *PRNP* as previously described (Malachin et al., 2017). Cells were allowed to grow for 48 h before stress exposure.

### Oxidative and genotoxic stress

PBMCs and SH-SY5Y cells were exposed to the alkylating agent methyl methanesulfonate (MMS, Sigma-Aldrich, Merck Life Science) (PBMCs: 0.5 mM; SH-SY5Y: 1.5 mM) and doxorubicin (Sigma-Aldrich, Merck Life Science) (PBMCs: 3 μM; SH-SY5Y: 2 μM) to induce DNA damage; methyl groups on nucleophilic sites of DNA bases and double-strand breaks (DSBs), respectively. To induce oxidative stress, cells were cultured with hydrogen peroxide (H_2_O_2_, Sigma-Aldrich, Merck Life Science) (PBMCs: 75 μM; SH-SY5Y: 150 μM). MMS was routinely removed from wells after 1 h and new culture media was added for recovery. Cells were incubated in 5% CO_2_, at 37°C, with their respective stressors for the designated amount of time.

### Viability of PBMCs

To analyze viability after stress exposure (H_2_O_2_, doxorubicin, MMS) in cells with or without PrP^C^, PBMCs (both genotypes: *n* = 4; 3 × 10^5^ cells/well), and SH-SY5Y cells (non-transfected and transfected cells: *n* = 3; 10^4^ cells/well) were cultured in a 96-well Greiner plate (Sigma-Aldrich, Merck Life Science) for a total of 24 h.

The average survival of the PBMCs from these four animals were included in the analysis. For SH-SY5Y cells, the experiment was repeated three times. Cell survival after 24 h was quantified using the Alamar Blue exclusion method and the fluorescence was read at 495 nm by Cytation 3 reader (BioTek Instruments, Winooski, VT).

To avoid the impact of differences in cell count, all stressed cells were compared to their own control, and expressed as percentage.

### *PRNP* transcriptional expression

PBMCs were added to 6-well plates (5 × 10^6^ cells/well) and exposed to MMS, H_2_O_2_, and doxorubicin, as described above. Cells were harvested after 1, 3, 6, and 24 h of exposure (1 h exposure with MMS, with recovery) and washed once with PBS containing 2 mM EDTA.

SH-SY5Y cells were plated and exposed in flasks (7.5 × 10^6^ cells/flask), followed by scraping in 1 ml of PBS.

All cell pellets were frozen in −70°C.

### RNA and DNA isolation, RT-qPCR

Total DNA was isolated from PBMCs and SH-SY5Y cells using a DNeasy Blood and tissue kit (Qiagen, Germantown, MD). Total RNA was isolated from frozen cell pellets using an RNeasy mini plus kit (Qiagen), following the manufacturer's instructions and quantified using a NanoDrop-1000 Spectrophotometer (ThermoFisher Scientific), prior to cDNA synthesis using SuperScript™-III reverse transcriptase, dNTPs mix, First-Strand Buffer, DTT, RNAse OUT™ (Invitrogen, ThermoFisher Scientific) and 500 ng of total RNA.

Quantitative PCR was performed using a LightCycler 480 Sybr Green I Master mix (Roche Holding AG, Basel, Switzerland) and run on a LightCycler 96 System (Roche), with the following primers *PRNP* (goat) F: GTGGCTACATGCTGGGAAGT, R: AGCCTGGGATTCTCTCTGGT; *PRNP* (human) F: CTGCTGGATGCTGGTTCTCT, R: GTGTTCCATCCTCCAGGCTT. See Malachin et al. for further details (Malachin et al., 2017).

### DNA damage analysis: LC-MS/MS quantification of 8-oxo(dG) and 7-m(dG)

DNA samples were digested by a mixture of nuclease P1 from *Penicillium citrinum* (N8630, Sigma-Aldrich, Merck Life Science), DNaseI (04716728001, Roche), and ALP from *E. coli* (P5931, Sigma-Aldrich, Merck Life Science) in 10 mM ammonium acetate buffer pH 5.3, 5 mM MgCl_2_, and 1 mM CaCl_2_ for 30 min at 40°C. The samples were methanol precipitated, supernatants were vacuum centrifuged at room temperature until dry, and dissolved in 50 μl of water for LC/MS/MS analysis. Quantification was performed with an LC-20AD HPLC system (Shimadzu, Kyoto, Japan) coupled to an AB Sciex API 5000 triple quadrupole (McKinley scientific, Sparta, NJ) operating in positive electrospray ionization mode. The chromatographic separation was performed with the use of a Supelco Ascentis Express C18 2.7 μm 150 × 2.1 mm i.d. column protected with a Supelco Ascentis Express Cartridge Guard Column (both from Ascentis, Sigma-Aldrich, Merck Life Science) with an Exp Titanium Hybrid Ferrule (Optimize Technologies Inc., Oregon City, OR). The mobile phase consisted of A (water, 0.1% formic acid) and B (methanol, 0.1% formic acid) solutions. The following conditions were employed for chromatography: for unmodified nucleosides—0.13 ml/min flow, starting at 10% B for 0.1 min, ramping to 60% B over 2.4 min, and re-equilibrating with 10% B for 4.5 min; for 8-oxo(dG)—0.14 ml/min flow, starting at 5% B for 0.5 min, ramping to 45% B over 8 min, and re-equilibrating with 5% B for 5.5 min. For mass spectrometry detection, the multiple reaction monitoring (MRM) was implemented using the following mass transitions: 252.2/136.1 (dA), 228.2/112.1 (dC), 268.2/152.1 (dG), 243.2/127.0 (dT), 284.1/168.1 [8-oxo(dG)].

### Transcriptomics

For transcriptomic analysis, PBMCs were harvested from 8 *PRNP*^+/+^ and 8 *PRNP*^Ter/Ter^ age-matched animals and RNA was isolated. RNA samples of high quality were shipped to Beijing Genomics Institute (BGI), Hong Kong. Paired-end sequencing was conducted on an Illumina HiSeq 2000 with 91 bp read-length, retrieving 5G clean data per sample. Raw data were analyzed using EdgeR (Robinson et al., 2010). For further details of the study protocol and analysis (see Malachin et al., 2017). All FASTQ files are available from the SRA database (SRA study accession number SRP102642).

Gene expression of enzymes involved in antioxidant defense and DNA damage repair were analyzed, specifically those involved in base excision repair (BER), nucleotide excision repair (NER), mismatch repair (MMR), and DSB repair.

### Statistical analysis

Statistical analyses were performed using GraphPad Prism version 6.07 (Graphpad, La Jolla, CA). Statistical significance was evaluated by multiple *t*-tests using the Holm-Sidak correction and *p* < 0.05 were regarded significant.

## Results

### PrP^c^ is abundantly expressed in the testicle and present on spermatozoa

**Figure 1:**
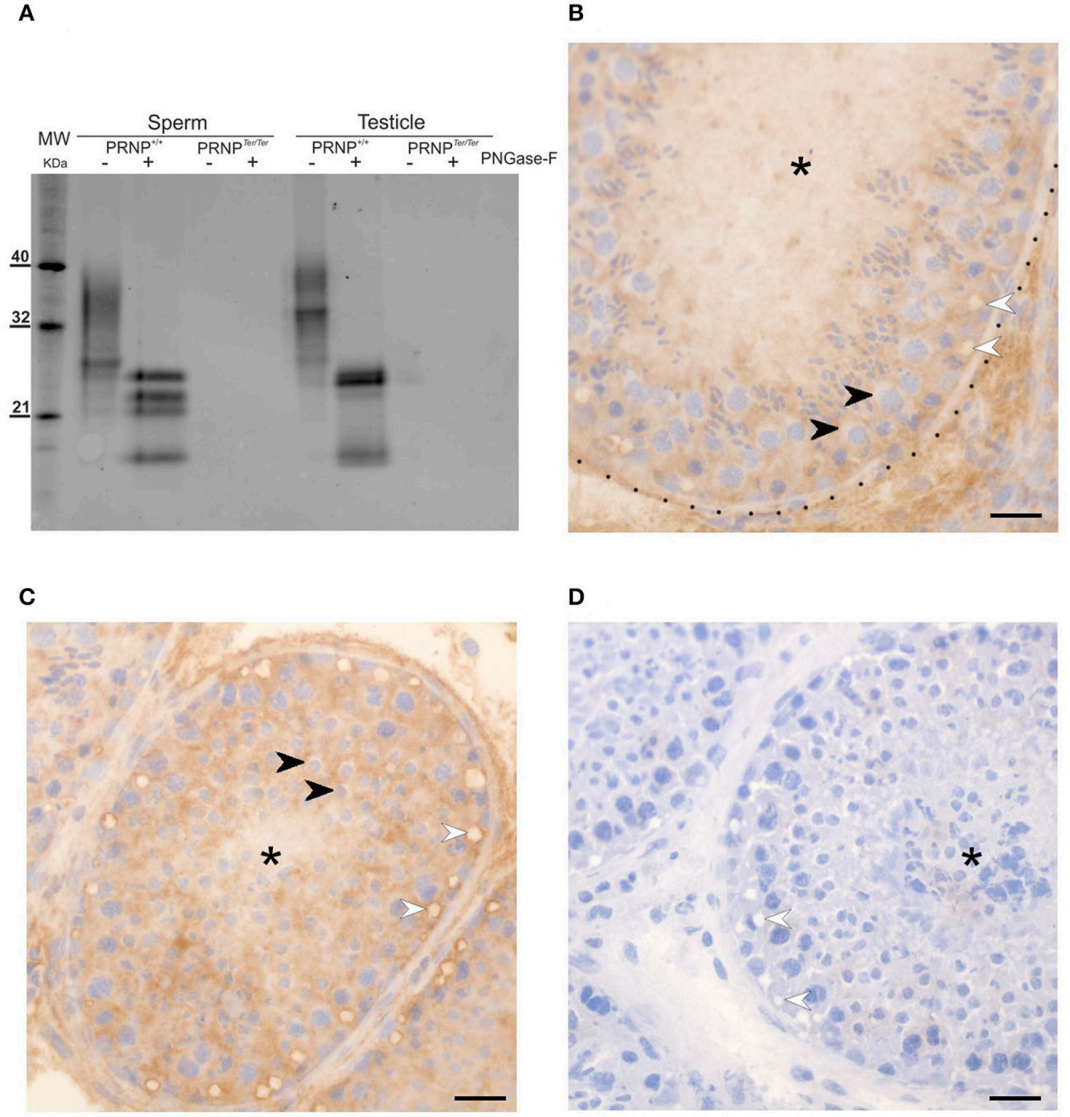
PrP^C^ is expressed in testicular tissue and spermatozoa. PrP^C^ was detected in testicle and spermatozoa by Western blot (WB) **(A)** and Immunohistochemistry (IHC) **(B–D)** using the P4 and 6H4 antibodies, respectively. In WB **(A)**, glycosylated full-length PrP^C^ is detected in both spermatozoa and testicular tissue. After deglycosylation with PNGaseF, several bands are visible on both preparations, including bands of approximately 25 and 18 kDa molecular mass, probably corresponding to full length PrP^C^ and a C-terminal fragment, respectively. Further bands with apparent molecular mass of 22–24 kDa are particularly prominent in samples from spermatozoa. No PrP^C^ could be detected in samples from *PRNP*^Ter/Ter^ animals. By IHC, a distinct PrP^C^ staining of seminiferous tubules with positive Sertoli cells, including the rim of vacuoles (white arrowheads) was observed. Spermatocytes **(B)** black arrowheads, and round spermatids **(C)** black arrowheads appeared negative for PrP^C^. Testicular tissue from *PRNP*^Ter/Ter^ bucks **(D)** are completely unstained, but similar in architecture and the presence of vacuoles (white arrowheads). The basement membrane of seminiferous tubules is indicated by dots, and the lumen by asterisk. (**B–D)**: x400. Scale bars 25 μm.

To evaluate the physiochemical properties of PrP^C^ in the testicle and spermatozoa, we performed Western Blot (WB) analysis from *PRNP*^+/+^ and *PRNP*^Ter/Ter^ bucks (Figure 1A, uncropped image available in Supplementary Materials). In *PRNP*^+/+^ samples not treated with PNGaseF, high molecular weight bands were present, apparently dominated by diglycosylated, full-length PrP^C^. Glycosylated PrP^C^ from testicle appeared heavier than in spermatozoa, probably reflecting differences in composition of the glycan moieties. After PNGase-F digestion, full length PrP^C^ at 27 kDa and a band of approximately 18 kDa were recovered from both spermatozoa and testicular tissue derived from the *PRNP*^+/+^ animals. The 18 kDa band corresponds well with a C-terminal fragment known as the C2 fragment derived after proteolytic processing of PrP^C^ in the octapeptide repeated sequence. Two further bands, particularly prominent in the preparations from spermatozoa with corresponding molecular masses at about 22–24 kDa, could represent PrP^C^ truncated from the C-terminus. Further studies are needed to clarify this.

**Figure 2:**
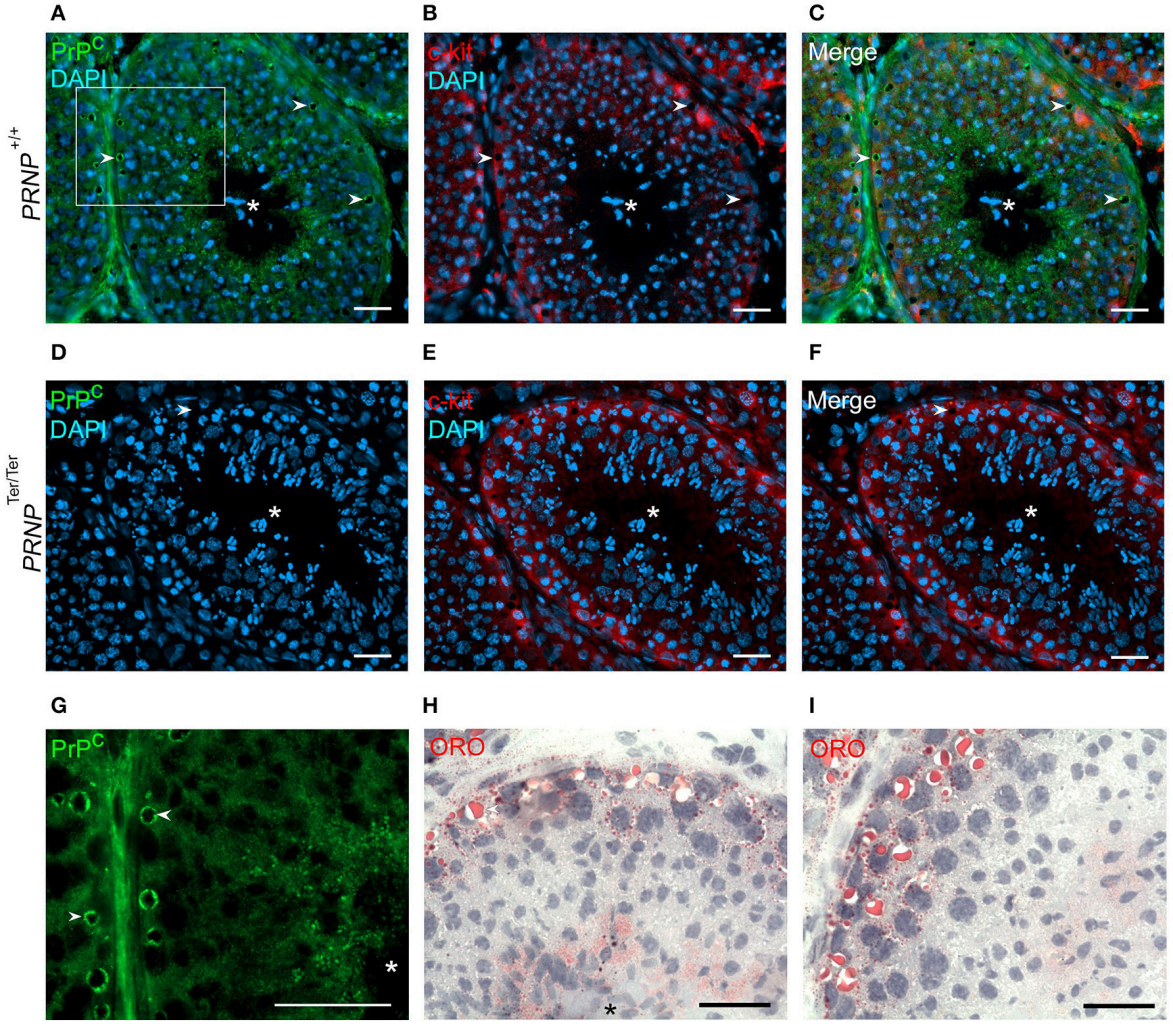
PrP^C^ is distinctly present in lipid vesicles and vacuoles in seminiferous tubules. Immunofluorescence analysis of testicular tissue demonstrates the presence of PrP^C^ **(A)** green, and the stem-cell marker c-kit **(B**) red, cell nuclei counterstained with DAPI (blue) and merged **(C)** in *PRNP*^+/+^ bucks. Spermatogenic stem cells positive for c-kit^+^ were distributed along the basement membrane and appeared to express low levels of PrP^C^. However, PrP^C^ was prominently present in small vesicles on the luminal aspect and in larger Sertoli cell vacuoles (arrowheads) along the periphery of the tubules, as highlighted by digital magnification **(G)** of rectangle depicted in **(A)**. Both the vesicles and vacuoles (arrowheads) contain lipids, as shown by ORO-staining **(H,I)**. Testis from *PRNP*^Ter/Ter^ bucks is negative for PrP^C^ and show similar distribution of c-kit and harbor similar vacuoles (arrowheads) as *PRNP*^+/+^ bucks **(D–F)**. Asterisk: Lumen of seminiferous tubules. x400 (all except G which was digitally magnified to x1,000). Scale bars 25 μm.

Analysis by IHC on testicles from *PRNP*^+/+^ bucks (Figures 1B,C) showed strong interstitial PrP^C^ staining in a pattern suggesting that both Leydig cells and connective tissue express PrP^C^. Along the basement membrane of the seminiferous tubules, a distinct PrP^C^-negative zone was noted. Inside the seminiferous tubules, PrP^C^ was distributed from the basement membrane through to the lumen in between the spermatogenic cell nuclei that in most developmental stages of the seminiferous cycle (França et al., 1999) were surrounded by a weakly stained or non-stained zone, indicating that the cytoplasm of the immature spermatogenic cells contains little PrP^C^. The staining pattern indicated that the Sertoli cells harbor PrP^C^ in their cytoplasm, including along the rim of peripherally located vacuoles and in projections toward the center of the tubules. PrP^C^ was not detected by IHC on tissue sections from *PRNP*^Ter/Ter^ bucks (Figure 1D). Immunofluorescent staining for PrP^C^ and c-kit (CD117) with DAPI for identification of nuclei (Figures 2A–C) confirmed the pattern of PrP^C^ distribution in the testicles of *PRNP*^+/+^ bucks. The c-kit^+^ spermatogonia close to the basement membrane showed weak PrP^C^ immunostaining, while the strongest signals were found in aggregates of small vesicles on the luminal side consistent with residual bodies; cytoplasmic droplets released from maturation phase spermatids and quickly engulfed by Sertoli cells. Immunolabeling was also seen through the tubular wall between cells to the basement membrane with the conspicuous peripheral PrP^C^-decorated vacuoles (Figure 2G), as also noted by IHC. Histochemical staining with ORO suggests that the PrP^C^-labeled vacuoles contain lipids (Figures 2H,I), described as a normal constituent of Sertoli cells (Wang et al., 2006). Lipid vacuoles were found at various stages. Interestingly, in association with the largest vacuoles, a significant number of small vesicles were present, some even outside the tubule in the basement membrane or the interstitial tissue. In stages of the spermatogenic cycle with abundant mature elongated spermatids, abundant small ORO-stained vesicles were located among the spermatids (Figure 2H), while after the release of the spermatids, the ORO staining toward the lumen was less prominent (Figure 2I). In *PRNP*^Ter/Ter^ bucks, PrP^C^ was not detected, whereas the c-kit labeling (Figures 2D–F) and ORO staining (data not shown) was similar as in the testicles of the *PRNP*^+/+^ bucks. The expression pattern of PrP^C^ in epididymis was established by IHC and IF (Supplementary Figure 1), both showing a strong staining of round basal cells within the epididymal epithelium and interstitial connective tissue. Smooth muscle cells stained weakly for PrP^C^. Tissues from *PRNP*^Ter/Ter^ bucks were negative by both methods. Global testicular levels of c-kit was assessed by WB and showed similar levels between the genotypes (Supplementary Figure 5 and uncropped image).

### Spermatozoa from *PRNP*^Ter/Ter^ and *PRNP*^+/+^ bucks show similar stress responses

**Figure 3:**
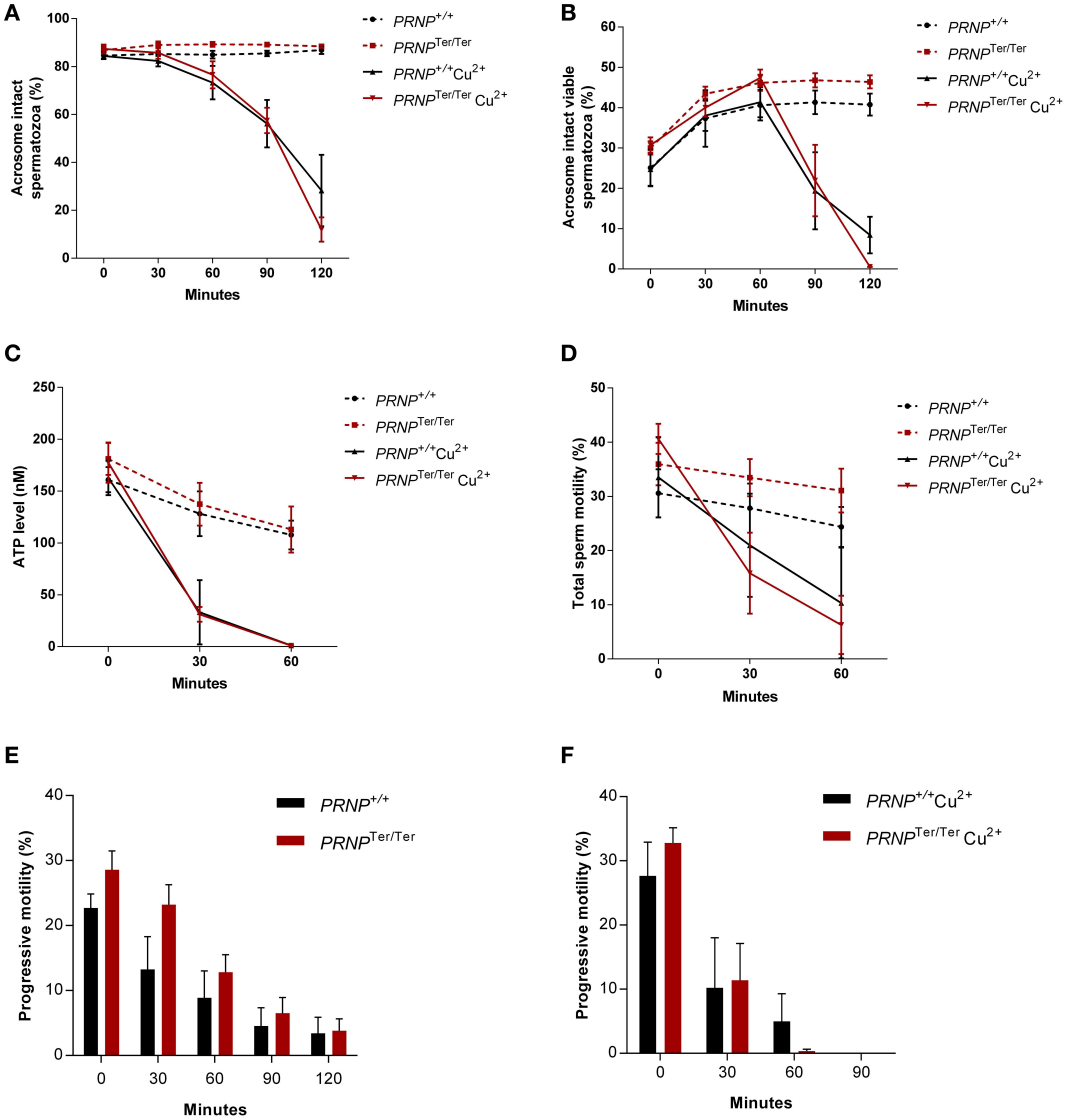
Expression of PrP^C^ does not increase viability upon induced oxidative stress in spermatozoa. Acrosome-intact spermatozoa **(A)**, acrosome-intact viable spermatozoa **(B)**, ATP levels **(C)**, total motility **(D)**, and progressive motility **(E,F)** were measured by flow cytometry in thawed spermatozoa from *PRNP*^+/+^ and *PRNP*^Ter/Ter^ bucks (both *n* = 4) at 0, 30, 60, 90, and 120 min after CuCl_2_-induced (100 μg/ml) oxidative stress. Values are shown as mean ± SEM (Significance tested by multiple *t*-test with Holm-Sidak correction).

To assess whether spermatozoa cells suffer from loss of PrP^C^ during an increase in ROS stress, we analyzed acrosome intactness, semen ATP levels and motility in CuCl_2_-treated and non-treated spermatozoa from *PRNP*^+/+^ and *PRNP*^Ter/Ter^ bucks (Figure 3). There was a clear dose-and time-dependent effect of CuCl_2_ in all experiments.

The acrosome intactness of non-treated spermatozoa did not differ between the genotypes and was consistent at ≈80% AI and ≈40% spermatozoa with intact acrosome live throughout the length of the experiment (Figures 3A,B). Treatment with 100 μg/ml CuCl_2_, on the other hand, resulted in a distinct decline in intact spermatozoa (Figure 3A). Importantly, there were no differences between the genotypes (Significance tested by multiple *t*-test with Holm-Sidak correction). In living spermatozoa, intactness seemed to increase during the first 30 min in both treated and non-treated cells, most likely due to a gradual decrease in permeability that stabilizes during incubation (Figure 3B).

Non-treated spermatozoa cells had a slow decline in ATP whereas CuCl_2_ treatment resulted in a rapid decline with ATP levels reaching zero after 60 min (Figure 3C).

Similar findings were observed after motility tests. Total and progressive motility from both genotypes declined during a 120 min interval (Figures 3D,E). The starting motility at time 0 was 30.6 and 36.0%, for *PRNP*^+/+^ and *PRNP*^Ter/Ter^, respectively. CuCl_2_ severely affected the spermatozoa total and progressive motility, which dropped to low levels after 60 min (100 μg/ml) (Figures 3D,F).

### ROS detection in spermatozoa

**Figure 4:**
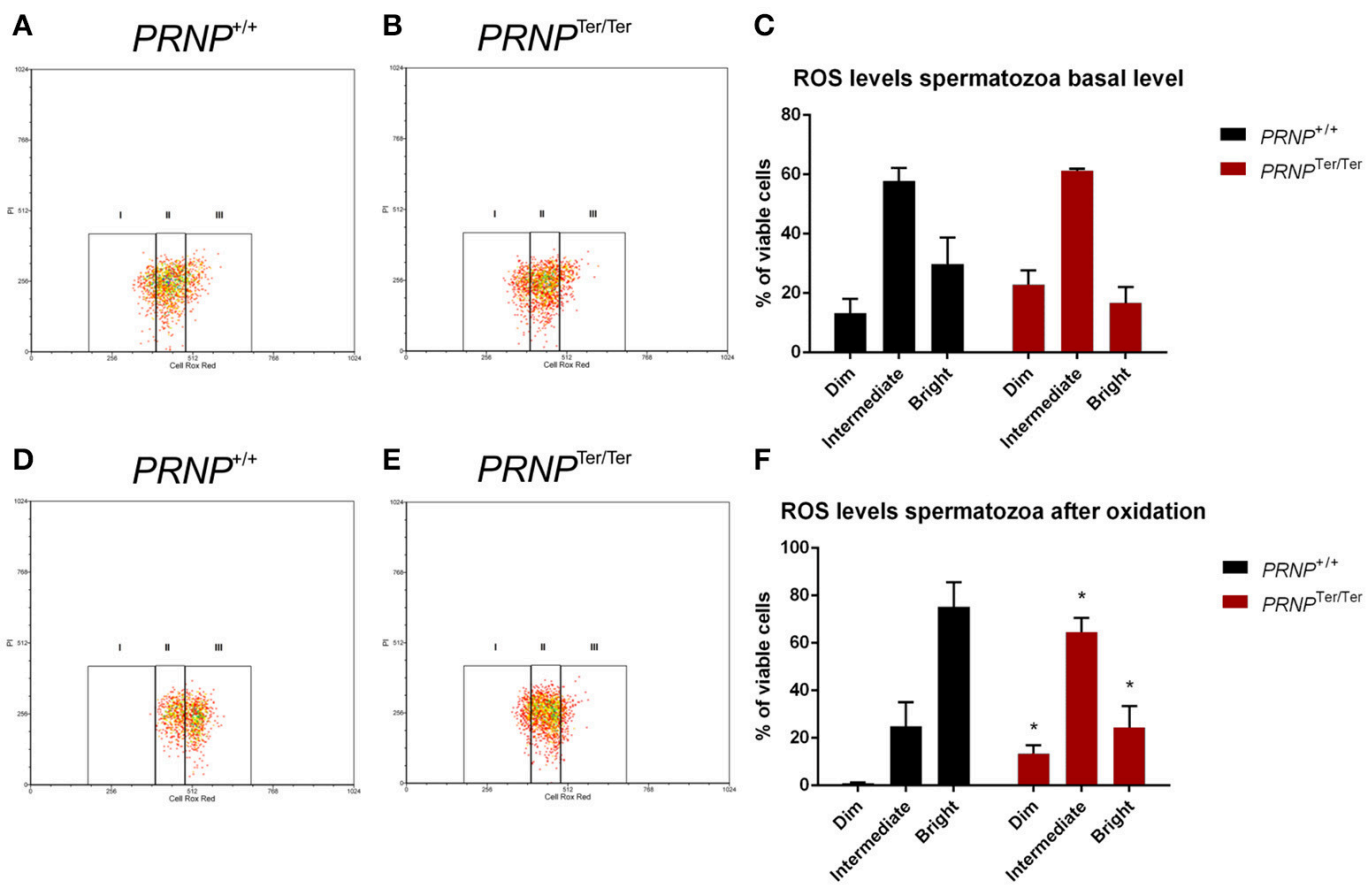
ROS levels in spermatozoa indicates no protection from PrP^C^. ROS levels were measured by flow cytometry in *PRNP*^+/+^ and *PRNP*^Ter/Ter^ spermatozoa after treatment with H_2_O_2_ and FeSO_4_ for 30 min. Plots from controls **(A,B)** and oxidized **(C,D)** spermatozoa are shown for both genotypes **(A**,**D)**: *PRNP*^+/+^, (**B**,**E)**: *PRNP*^Ter/Ter^. Based on fluorescence intensity, spermatozoa were gated into regions dim, intermediate and bright, of which region bright represents spermatozoa with the highest ROS levels. Mean basal **(C)** ROS levels and mean ROS levels after H_2_O_2_ and FeSO_4_ treatment **(F)** in both genotypes is shown (*n* = 4). The percentages of viable cells before ROS exposure were 39.19 ± 4.98 and 37.75 ± 3.67 for *PRNP*^+/+^ and *PRNP*^Ter/Ter^, respectively. The corresponding values after ROS exposure were 39.66 ± 3.69 (*PRNP*^+/+^) and 35.58 ± 3.67 (*PRNP*^Ter/Ter^). Values are shown as mean ± SEM. ^*^indicates *p* < 0.05 (Significance tested by multiple *t*-test with Holm-Sidak correction).

To evaluate whether spermatozoa without PrP^C^ react differently to oxidative stress induced by H_2_O_2_ in combination with FeSO_4_, levels of ROS were assessed by flow cytometry using CRR prior to and after oxidation (Figure 4). Incubating spermatozoa for 30 min with 500 μM FeSO_4_·7H_2_O and 196 μM H_2_O_2_ did not induce cell death as there were no differences in viability before and after exposure. Before ROS exposure, the percentage of viable cells was 39.19 ± 4.98 in the *PRNP*^+/+^ group and 37.75 ± 3.65 for the *PRNP*^Ter/Ter^ animals. The corresponding values after ROS exposure were 39.66 ± 3.69 and 35.58 ± 3.67 for the *PRNP*^+/+^ and *PRNP*^Ter/Ter^ group respectively (*n* = 4, all groups). Viable cells were discriminated according to accumulated ROS levels; dim, intermediate or bright. A shift in fluorescence signal reflecting different levels of ROS in *PRNP*^+/+^ vs. *PRNP*^Ter/Ter^ spermatozoa was detected in all regions (Figures 4C,F). Approximately 75% of the *PRNP*^+/+^ spermatozoa fell into the bright region, indicating high ROS levels, as compared to only 24.38% of the *PRNP*^Ter/Ter^ spermatozoa, after induction of oxidative stress. The results indicate that *PRNP*^+/+^ spermatozoa accumulated higher levels of ROS than *PRNP*^Ter/Ter^ cells but even so, the viability was similar between the genotypes.

### Viability of peripheral blood mononuclear cells with and without PrP^c^ expression upon cellular stress exposure

**Figure 5:**
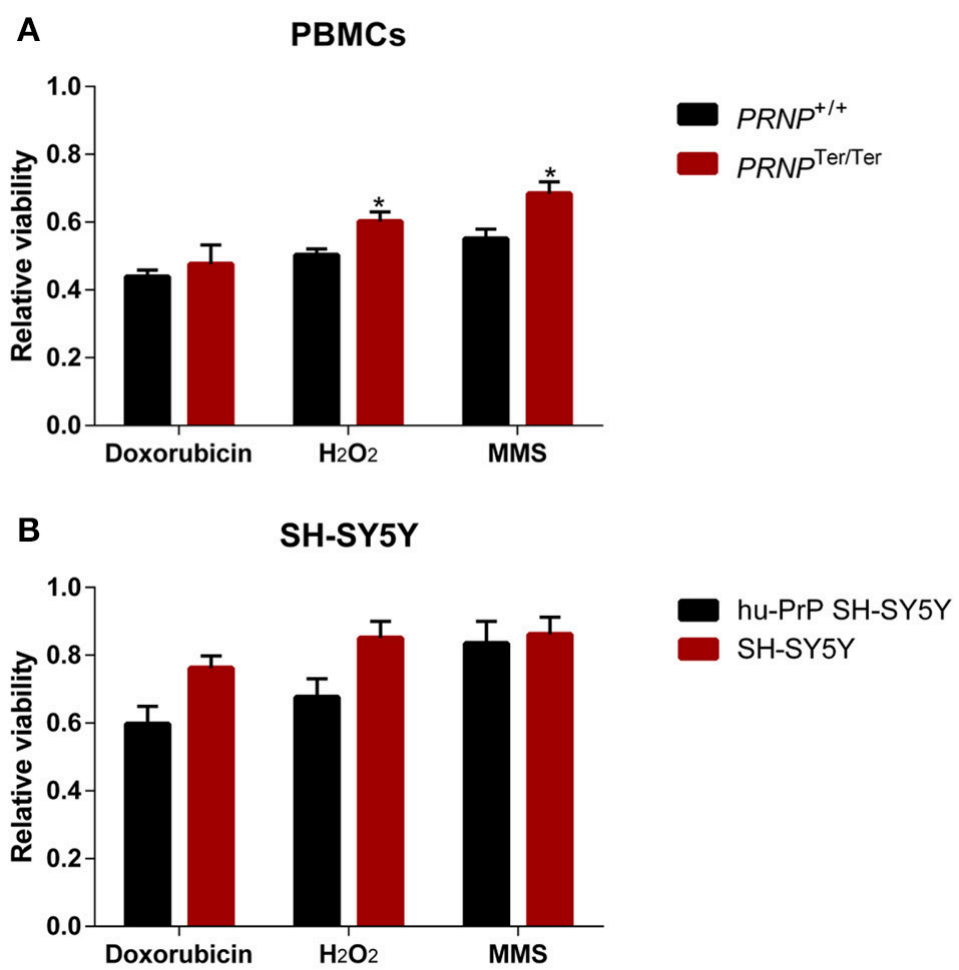
Lack of PrP^C^ expression does not decrease cellular viability. Peripheral blood mononuclear cells (PBMCs) with and without PrP^C^ expression, and human neuroblastoma SH-SY5Y cells with very low PrP^C^ levels (mock-transfected) and moderate PrP^C^ levels (stably transfected with human PrP^C^), were treated for 24 h with doxorubicin (PBMCs 3 μM, SH-SY5Y 2 μM) or H_2_O_2_ (PBMCs 75 μM, SH-SY5Y 150 μM), or methyl methanesulfonate (MMS) (PBMCs 0.5 mM, SH-SY5Y 1.5 mM) for 1 h, with 23 h recovery. Viability in PBMCs (*n* = 4) **(A)** and SH-SY5Y cells (*n* = 3) **(B)** (relative to controls) with and without PrP^C^ expression after induction of cellular stress with doxorubicin, H_2_O_2_ and MMS is shown, assessed by the Alamar Blue assay using Cytation 3. Values are given as mean ± SEM. ^*^ indicates *p* < 0.05 (Significance tested by multiple *t*-test with Holm-Sidak correction).

Considering that spermatozoa cells are highly specialized cells expressing only limited sets of proteins, we decided to include other cell types in the study of PrP^C^'s role in *in vitro* stress resilience. Thus, we measured the relative viability 24 h after treatment with MMS (1 h with 23 h recovery), H_2_O_2_ and doxorubicin (both 24 h treatment) in primary PBMCs from both genotypes, and human neuroblastoma cell line SH-SY5Y cells and hu-PrP SH-SY5Y cells, stably expressing moderate (80- to 100-fold, mRNA) higher levels of PrP^C^ than un-transfected SH-SY5Y cells, which express very low levels of PrP^C^. Human neuroblastoma cells were included for analysis of PrP-associated phenotypes in a neuronal cell line, different from goat immune cells and spermatozoa. Surprisingly, viability was significantly higher in *PRNP*^Ter/Ter^ PBMCs after both H_2_O_2_ and MMS exposure (Figure 5A). With doxorubicin, PBMCs from both genotypes reacted similarly. No significant differences were detected between SH-SY5Y and hu-PrP SH-SY5Y cells (Figure 5B). The *PRNP* mRNA expression levels in PBMCs increased slightly after doxorubicin treatment (Supplementary Figure 2A); however, after 24 h of doxorubicin, the expression was downregulated. No major regulation of *PRNP* expression was observed with the other stressors. In both hu-PrP SH-SY5Y and SH-SY5Y cells, a consistent but moderate upregulation of *PRNP* expression was observed with all stressors (Supplementary Figures 2B–C).

### No differences in DNA damage levels after oxidative and genotoxic stress

**Figure 6:**
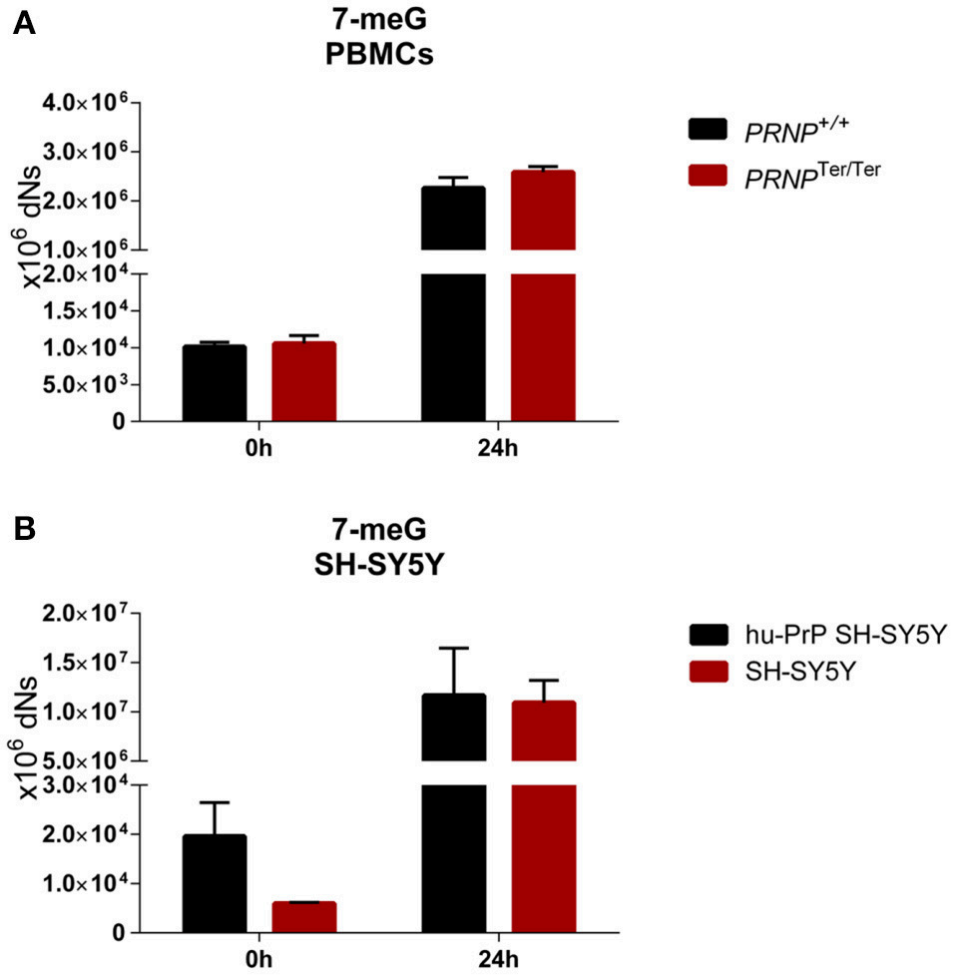
Similar accumulation of the methyl adduct 7-meG in PBMCs and SH-SY5Y cells with and without PrP^C^ expression after treatment with MMS. PBMCs (*n* = 3–5) **(A)** and human neuroblastoma SH-SY5Y cells (*n* = 2) **(B)** with and without PrP^C^ expression were exposed to MMS for 1 h, with 23 h recovery. Levels of 7-meG were assessed 24 h after exposure. Data are given as mean ± SEM (Significance tested by multiple *t*-test with Holm-Sidak correction, all *p* > 0.05).

The 7-m(dG) lesions induced by MMS exposure increased dramatically after MMS treatment in both cell types. Importantly, there were no differences between the genotypes (Figure 6). The accumulation of lesions was most profoundly seen in SH-SY5Y cells, although the levels of 7-m(dG) after 1 h (data not shown) and 24 h were similar, indicating that the cells had reached a maximum lesion threshold already after 1 h.

H_2_O_2_-induced oxidative stress yielded a similar amount of 8-oxoG lesions in both genotypes (Supplementary Figure 3), indicating that the presence of PrP^C^ is not essential to maintain the DNA damage-repair response. Notably, in PBMCs, the major enzymes involved in base-excision repair (BER), nucleotide-excision repair (NER), DSB repair, and MMR displayed no significant difference in mRNA expression levels between the two genotypes (Supplementary Figures 4A–D). No differences were detected in mRNA expression levels for antioxidant enzymes either (Supplementary Figure 4E).

## Discussion

The expanding literature dealing with putative physiological functions of PrP^C^ has recently been comprehensively reviewed (Castle and Gill, 2017; Linden, 2017; Wulf et al., 2017). Some of the methodological challenges inherent to various animal models, particularly concerning genetic confounders have also been elaborated (Steele et al., 2007). Here, we have used an alternative animal model for prion research; dairy goats that completely lack PrP^C^, caused by a nonsense mutation affecting codon 32 in the PrP^C^ reading frame (Benestad et al., 2012).

The potential value of goats without PrP^C^ for breeding purposes, specifically to combat scrapie in goats, or for the production of “prion free” bio-products depends not only on the production parameters, general health and fitness of the animals, but also their fertility and reproductive capacity in breeding systems using artificial insemination (AI) with frozen semen. In view of data from mice demonstrating that PrP^C^ significantly protected spermatozoa against metal (Cu^2+^)-induced oxidative stress (Shaked et al., 1999), we assumed that spermatozoa from bucks homozygous for the *PRNP*^Ter^ mutation would display increased stress sensitivity possibly leading to reduced viability after storage in liquid nitrogen. We have observed normal offspring after natural breeding of homozygous *PRNP*^Ter/Ter^ bucks and goats, providing evidence that lack of PrP^C^ does not inflict a major fertility problem for the goats, which is accordance with data from *PRNP* KO mice. Moreover, during the course of this work we have performed one AI mating with semen from a *PRNP*^Ter/Ter^ buck with a goat of the same genotype and received normal offspring, indicating intact male fertility also after cry-preservation of semen.

Analysis of ejaculated spermatozoa and samples from testicles with WB demonstrated that *PRNP*^Ter/Ter^ bucks completely lack PrP^C^, whereas *PRNP*^+/+^ bucks show a significant presence of PrP^C^. The protein is predominantly di-glycosylated, with somewhat heavier molecular masses in the testicle as compared with spermatozoa preparations. Upon deglycosylation, full length PrP^C^ (27 kDa) and a band with an estimated molecular mass that corresponds to the C2 cleavage product (18 kDa) of PrP^C^, are recovered from both preparations. The C2 fragment stems from ß-cleavage of PrP^C^, known to be stimulated by oxidative stress, probably cleaving goat PrP^C^ between His88 and Gly89 (McDonald et al., 2014). Since the mab used here (P4) binds to amino acids 95–105 (Harmeyer et al., 1998), it detects the C2 fragment of PrP^C^, but not the C1 fragment generated by α-cleavage, which most likely occurs around amino acid 115 in goat PrP^C^ (Chen et al., 1995; Tveit et al., 2005). If the 18 kDa band shown here stems from ß-cleavage of PrP^C^, it could indicate that this cleavage occurs more frequently in the testicle and spermatozoa, compared to brain, in which the C2 fragment is at low levels. Moreover, it has been shown that PrP^C^ can be liberated from the cell membrane by cleavage close to the C-terminus, generating a protein species about 4–6 kDa smaller than full length PrP^C^ due to the loss of the GPI anchor (Taylor et al., 2009; McDonald et al., 2014). This might be of relevance in interpreting the two distinct bands with molecular mass between 22 and 24 kDa observed in preparations from spermatozoa. However, as demonstrated in several previous studies (Shaked et al., 1999; Ecroyd et al., 2004), detailed epitope mapping of PrP^C^ species from the male genital organs and spermatozoa is challenging, and conflicting data have emerged. Although interesting, solving this task was outside the scope of the current investigations.

By IHC and IF, PrP^C^ was found most abundantly in Sertoli cells, including in large cytoplasmic Oil-Red-O (ORO)-positive lipid vacuoles, particularly prominent in the periphery of the seminiferous tubules. Ford et al. (2002) showed that an intense PrP^C^-positive immunostaining could be detected in lipid droplets shed as residual bodies from the spermatozoa as part of their maturation process. These residual bodies are phagocytosed by the Sertoli cells and transformed into small phagolysosomal, ORO-positive lipid vesicles (Wang et al., 2006), as found along the luminal aspects of the tubules in the present study. As the distribution of ORO-positive residual bodies seem to overlap with the granular PrP^C^-staining, it is likely that the residual bodies contain PrP^C^. Whether PrP^C^ in residual bodies stems from the phagocytosed spermatid-released cytoplasmic droplets, produced by the Sertoli cell itself, or a combination of the two, remains to be investigated. The large lipid vacuoles in the basal aspect of the Sertoli cells were present in almost all stages of the goat spermatogenic cycle (Onyango et al., 2000) and may stem from the degradation of apoptotic spermatogenic cells (Wang et al., 2006) and/or residual bodies (Paniagua et al., 1987). The latter structures were positive for PrP^C^ and ORO, and given that the spermatogenic cells were mostly PrP^C^ negative, a co-transport of PrP^C^ and lipids from the residual body stage to the larger vacuoles seems possible. Smaller vacuoles, maybe from degradation of the larger vacuoles, were observed to disseminate across the basement membrane, similar to the way immunogenic antigens are transported (Krawetz et al., 2009). Studies have found *PRNP* mRNA in several developmental stages of spermatozoa and in Sertoli cells (Ford et al., 2002; Fujisawa et al., 2004).

PrP^C^ has been detected in the Sertoli cells of immature goat bucks (130 days post conception) and in ejaculated buck spermatozoa (Allais-Bonnet et al., 2016). Levels of *PRNP* mRNA were high in buck testicular tissue from birth and gradually decreased to reach a steady level at puberty. This observation might suggest that PrP^C^ in the testicle serves roles that are not specific to spermatogenesis. This is in contrast to the prion-like protein Doppel (Dpl), encoded by *Prnd*, which is expressed at low levels in sexually immature bucks, before raising sharply in expression toward puberty, after which it remains at a high level (Peoc'h et al., 2002; Espenes et al., 2006; Kocer et al., 2007). Interestingly, genetic knockout of *Prnd* renders male mice infertile (Behrens et al., 2002), whereas absence of PrP^C^ apparently has no direct effect on male fertility, neither in mice nor in goat bucks (Bueler et al., 1992).

It has been proposed that PrP^C^ is present in spermatocytes and spermatids; although absent in the earlier spermatogonia (Peoc'h et al., 2002). Our data demonstrate high PrP^C^ levels in Sertoli cells, raising the intriguing possibility that PrP^C^ on spermatids could originate from the Sertoli cells. Studies of PrP^C^ in spermatozoa and in fluids along the ram genital tract revealed that the epididymal fluid contained significant levels of what appeared to be soluble, highly glycosylated forms of the prion protein (Gatti et al., 2002). The authors proposed that some of PrP^C^ species present on ejaculated spermatozoa could be acquired from the seminal fluid during ejaculation. While our investigations have not focused on seminal plasma or epididymal fluids, IHC and IF analysis of the epididymis showed that PrP^C^ is present in cells below the columnar epithelium, which itself appeared negative. However, the inter-tubular connective tissue was strongly positive for PrP^C^, whereas the smooth muscle stained substantially weaker for PrP^C^. Although, these observations do not rule out secretion of PrP^C^ from the epididymal epithelium, it clearly illustrates that PrP^C^ appears to serve functions in the epididymis that are unrelated to production and release of PrP^C^ into the seminal fluid.

Analyses of acrosome intactness, viability, ATP levels and motility after oxidative stress induction with CuCl_2_ showed no differences between the genotypes in their ability to handle this stressor. The dramatic reduction in parameters are in line with other studies on oxidative stress in spermatozoa (Koppers et al., 2008). Our finding differ from results reported by Shaked et al. (1999); namely that Cu^2+^ exposure caused motility loss at a faster rate in spermatozoa from *Prnp* KO mice. This apparent discrepancy could be due to different experimental procedures or represent true species differences. For instance, Shaked et al. studied spermatozoa extracted from the epididymis, whereas we studied ejaculated spermatozoa that have undergone routine dilutions and freezing in liquid nitrogen. Epididymal mouse spermatozoa are immobile, but motility can be established by dilution in specific buffers (Tash and Bracho, 1998). In our preparations of spermatozoa that had undergone cryopreservation, approximately 30–40% of the spermatozoa were recorded as motile. We observed, as expected, a gradual decline in ATP levels and motility in untreated controls for both genotype groups during the 60 min incubation. From a breeder's point of view, it can be concluded that all fundamental spermatozoa parameters as recorded in cryopreserved semen, appear unaffected by the loss of PrP^C^ in the goat buck.

In order to explore further *in vitro* stress resilience, we analyzed accumulations of ROS in spermatozoa from *PRNP*^+/+^ and *PRNP*^Ter/Ter^ bucks. Interestingly, we observed that a larger proportion of viable spermatozoa from *PRNP*^+/+^ bucks fell into the “high ROS” category (bright), demonstrating that under these experimental conditions, higher numbers of PrP^C^-containing spermatozoa contained higher ROS levels, while still remaining viable. This surprising observation indicates that in mature spermatozoa, PrP^C^ appears not to contribute to ROS scavenging capacity. This is in contrast to previous observations of increased levels of ROS in various diploid PrP^C^-deficient cell lines (Choi et al., 2007; Aude-Garcia et al., 2011; Bertuchi et al., 2012; Zanetti et al., 2014).

We wanted to test whether our observation of complete lack of PrP^C^-mediated stress protection could be a peculiarity of spermatozoa prepared for AI. In order to achieve this, we exposed goat PBMCs with and without PrP^C^ and human neuroblastoma SH-SY5Y cells expressing different levels of PrP^C^ to different types of genotoxic and oxidative stress. Interestingly, upon exposure of cells to doxorubicin, an inducer of DNA double strand breaks, H_2_O_2_ for induction of ROS stress, and MMS for introduction of methyl adducts, we were unable to detect any stress-protective effect of PrP^C^ in terms of cellular viability in any of the cell types. Contrary, we observed a statistically significant increase in relative viability of PBMCs derived from *PRNP*^Ter/Ter^ animals after H_2_O_2_ and MMS treatment, compared with PBMCs from *PRNP*^+/+^ animals. The biological significance or molecular explanation of this surprising observation remain to be clarified. There was no effect of PrP^C^ on levels of 7-m(dG), neither before nor after treatment with MMS. Our data appear to be in line with the conclusion drawn by Castle and Gill (Castle and Gill, 2017), in their recent review, that a direct stress-protective function for PrP^C^ remains unproven. In accordance with previous observations, the SH-SY5Y cells increased their expression of endogenous PrP^C^ in response to severe stress (Bravard et al., 2015). This effect was less consistent in PBMCs, although a clear induction was evident after treatment with doxorubicin. Our data do not provide support for the concept that PrP^C^ is critically important for DNA repair, as reported by Bravard et al. (2015). They observed decreased survival of SH-SY5Y cells without PrP^C^ after treatment with MMS and that DNA lesions were increased in the absence of PrP^C^. Further investigations are needed to clarify this issue.

Taken together, our data, derived from different cell types and under a variety of stressful conditions, show that *in vitro* there appears to be no stress-protective effects of PrP^C^. The findings are in contrast to studies that have shown that PrP^C^ can positively influence survival of cells exposed to xanthine oxidase (Brown et al., 1997, 2002), H_2_O_2_ (White et al., 1999; Oh et al., 2012), and paraquat (Senator et al., 2004; Dupiereux et al., 2008). It has been suggested that antioxidant enzyme activities such as glutathione reductase (White et al., 1999) or SOD-1 (Brown et al., 1997) are reduced in the absence of PrP^C^. Other studies have not found changes in the enzyme activities of glutathione peroxidase, catalase, and Cu/Zn SOD (Brown et al., 2002). It has also been proposed that PrP^C^ stimulates the expression of antioxidant enzymes (White et al., 1999; Klamt et al., 2001) and that this could partly explain the protective effect of PrP^C^ against oxidative stress. However, RNA sequencing data from PBMCs derived from goats with or without PrP^C^ do not support this notion, since we were unable to detect any differences between genotypes in expression of major DNA repair enzymes or a panel of enzymatic antioxidants.

Interestingly, increased levels of oxidative DNA damage have been detected in cells without PrP^C^ during normal physiological states (Watt et al., 2007). Higher levels of oxidated lipids and proteins were reported in the CNS (Wong et al., 2001; bio Klamt et al., 2001) and peripheral structures (bio Klamt et al., 2001) of *Prnp* KO mice, reflecting a higher oxidative load. These scenarios are likely to have yielded higher levels of enzymes involved in these repair processes (Vogel and Marcotte, 2012); however, we were unable to detect any major differences in neither DNA damage-repair enzymes nor enzymatic antioxidants in PBMCs from *PRNP*^+/+^ and *PRNP*^Ter/Ter^ animals.

In conclusion, our observations of PBMCs, spermatozoa and SH-SY5Y cells after induction of different forms of severe cellular stress suggest that PrP^C^ is not directly protective against these stressors *in vitro*. This, however, does not rule out that PrP^C^ could serve protective functions *in vivo*, particularly during inflammation, as suggested in several studies in mice (McLennan et al., 2004; Martin et al., 2011; Gourdain et al., 2012; Ezpeleta et al., 2017), and goats (Salvesen et al., 2016, 2017).

## Ethics statement

This study was carried out in accordance with the recommendations of the Norwegian regulation on animal experimentation, FOR-2015-06-18-761. The protocol was approved by the Committee on the Ethics of Animal Experiments by The Norwegian Animal Research Authority (ID No. 8058).

## Author contributions

MR, GM, EK, KW, AKK, MB, MKB, AE, and MT: designed the experiments; MR, GM, GØ, KW, AK, CJ, LN, SR, E-BS, FM, TZ, and MKB: conducted the experiments; MR, GM, EK, KW, AKK, MB, MKB, AE, and MT: analyzed the data; MR, GM, EK, KW, MKB, AE, and MT: wrote the paper. All authors read and approved the final manuscript.

### Conflict of interest statement

KW was employed by the company Spermvital AS, Holsetgata. The other authors declare that the research was conducted in the absence of any commercial or financial relationships that could be construed as a potential conflict of interest.
