# Supplementary material for: Stress Resilience of Spermatozoa and Blood Mononuclear Cells without Prion Protein
Source: Front Mol Biosci. 2018 Jan 24;5:1. doi: 10.3389/fmolb.2018.00001 (PMC5787566; doi:10.3389/fmolb.2018.00001)
Supplement: Supplementary file 1 [file Image1.PDF]

frontiers

Supplementary Material

Stress resilience of spermatozoa and blood mononuclear cells without prion protein

Malin R. Reiten[1], Giulia Malachin[1], Elisabeth Kommisrud[2], Gunn C. Østby[1], Karin E. Waterhouse[1,4], Anette K. Krogenæs[1], Anna Kusnierczyk[3], Magnar Bjørås[3], Clara M. O. Jalland[1], Liv Heidi Nekså[1], Susan S. Røed[1], Else-Berit Stenseth[2], Frøydis D. Myromslien[2], Teklu T. Zeremichael[2], Maren K. Bakkebø[1], Arild Espenes[1], Michael A. Tranulis[1*]

[1]Faculty of Veterinary Medicine and Biosciences, Norwegian University of Life Sciences, Oslo, Norway

2 Faculty of Education and Natural Sciences, Inland University of Applied Sciences, Hamar, Norway

[3]Department of Cancer Research and Molecular Medicine, Norwegian University of Science and Technology, Trondheim, Norway

[4]Spermvital AS Holsetgata 22, Hamar, Norway

*** Correspondence:**
Michael A. Tranulis
michael.tranulis@nmbu.no

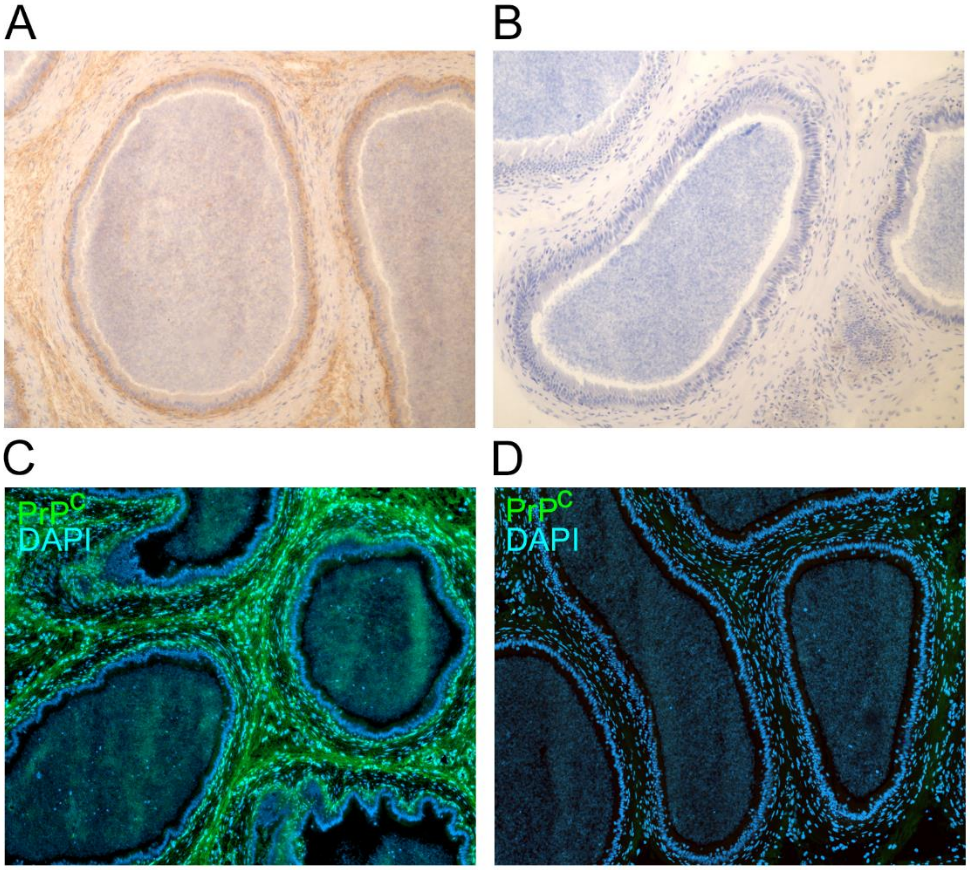

Supplementary Figure 1: PrP^{C} is expressed in epididymis

PrP^{C} expression in epididymis was detected by IHC (A and B) and IF (C and D) using the 6H4 antibody. IHC of *PRNP*$^{+/+}$ (A) tissue showed a strong staining of PrP^{C} in the layer of basal cells below the columnar epithelium lining the epididymal duct. Strong staining was detected also in the interstitial connective tissue whereas the smooth muscle cells exhibited a weak PrP^{C} staining. IF confirmed the staining pattern (C), with PrP^{C} (green) and DAPI (blue). *PRNP*$^{Ter/Ter}$ tissues were negative both by IHC (B) and IF (D).
