# Supplementary material for: Stress Resilience of Spermatozoa and Blood Mononuclear Cells without Prion Protein
Source: Front Mol Biosci. 2018 Jan 24;5:1. doi: 10.3389/fmolb.2018.00001 (PMC5787566; doi:10.3389/fmolb.2018.00001)
Supplement: Supplementary file 2 [file Image2.PDF]

Supplementary Material

Stress resilience of spermatozoa and blood mononuclear cells without prion protein

Malin R. Reiten[1], Giulia Malachin[1], Elisabeth Kommisrud[2], Gunn C. Østby[1], Karin E. Waterhouse[1,4], Anette K. Krogenæs[1], Anna Kusnierczyk[3], Magnar Bjørås[3], Clara M. O. Jalland[1], Liv Heidi Nekså[1], Susan S. Røed[1], Else-Berit Stenseth[2], Frøydis D. Myromslien[2], Teklu T. Zeremichael[2], Maren K. Bakkebø[1], Arild Espenes[1], Michael A. Tranulis[1*]

[1]Faculty of Veterinary Medicine and Biosciences, Norwegian University of Life Sciences, Oslo, Norway

2 Faculty of Education and Natural Sciences, Inland University of Applied Sciences, Hamar, Norway

[3]Department of Cancer Research and Molecular Medicine, Norwegian University of Science and Technology, Trondheim, Norway

[4]Spermvital AS Holsetgata 22, Hamar, Norway

*** Correspondence:**
Michael A. Tranulis
michael.tranulis@nmbu.no

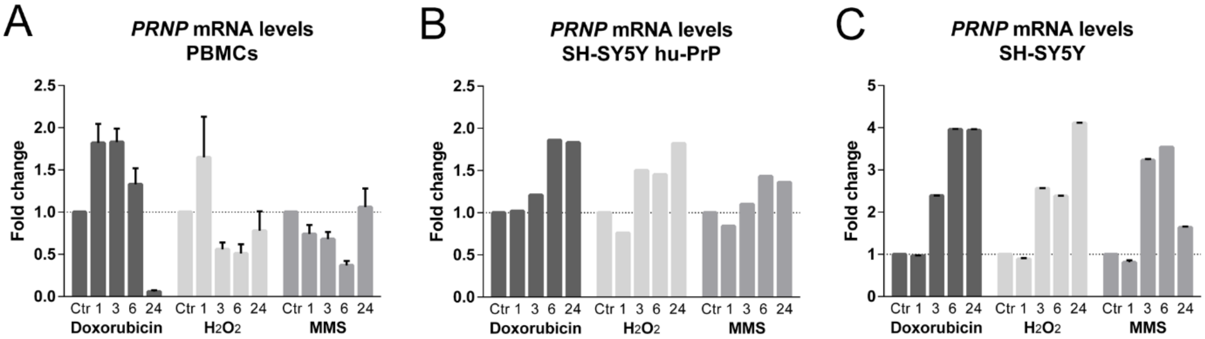

Supplementary Figure 2: Minor upregulation of *PRNP* after cellular stress in SH-SY5Y cells
Peripheral blood mononuclear cells (PBMCs) and human neuroblastoma SH-SY5Y cells with and without PrP^C expression were incubated with doxorubicin or H_2O_2 for 1, 3, 6 and 24 hrs, or with MMS for 1 h, with 0, 2, 5 and 23 hrs recovery. *PRNP* mRNA levels in PBMCs (n = 3-4) (A), Hu-PrP SH-SY5Y cells (n = 4) (B) and SH-SY5Y cells (n = 4) (C), measured in controls and at 1, 3, 6 and 24 hrs after treatment, are shown as fold change compared to control cells.

Values are shown as mean ± SEM. (Significance tested by multiple t-test with Holm-Sidak correction, all p-values > 0.05).
