# Supplementary material for: Stress Resilience of Spermatozoa and Blood Mononuclear Cells without Prion Protein
Source: Front Mol Biosci. 2018 Jan 24;5:1. doi: 10.3389/fmolb.2018.00001 (PMC5787566; doi:10.3389/fmolb.2018.00001)
Supplement: Supplementary file 3 [file Image3.PDF]

Supplementary Material

Stress resilience of spermatozoa and blood mononuclear cells without prion protein

Malin R. Reiten[1], Giulia Malachin[1], Elisabeth Kommisrud[2], Gunn C. Østby[1], Karin E. Waterhouse[1,4], Anette K. Krogenæs[1], Anna Kusnierczyk[3], Magnar Bjørås[3], Clara M. O. Jalland[1], Liv Heidi Nekså[1], Susan S. Røed[1], Else-Berit Stenseth[2], Frøydis D. Myromslien[2], Teklu T. Zeremichael[2], Maren K. Bakkebø[1], Arild Espenes[1], Michael A. Tranulis[1*]

[1]Faculty of Veterinary Medicine and Biosciences, Norwegian University of Life Sciences, Oslo, Norway

2 Faculty of Education and Natural Sciences, Inland University of Applied Sciences, Hamar, Norway

[3]Department of Cancer Research and Molecular Medicine, Norwegian University of Science and Technology, Trondheim, Norway

[4]Spermvital AS Holsetgata 22, Hamar, Norway

*** Correspondence:**
Michael A. Tranulis
michael.tranulis@nmbu.no

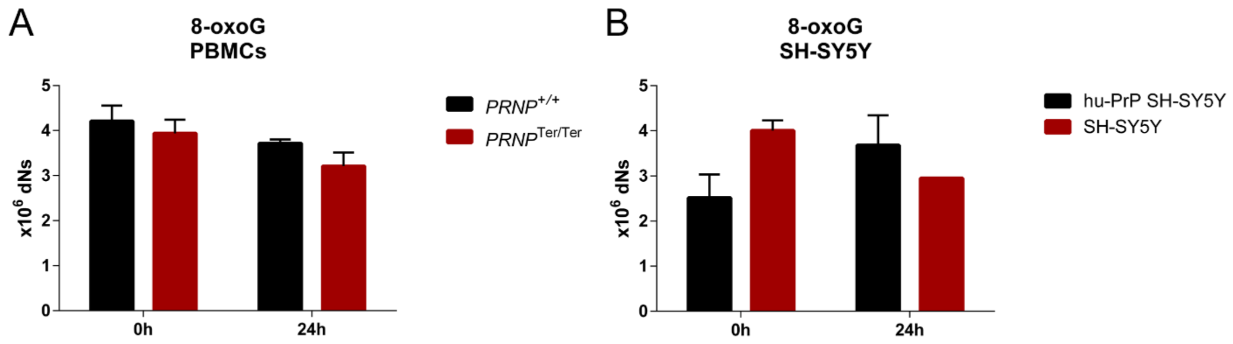

Supplementary Figure 3: H_2O_2-induced oxidative stress creates equal amounts of 8-oxoG lesions in PBMCs and SH-SY5Y cells with and without PrP^C expression
PBMCs (*n* = 3-5) (A) and SH-SY5Y cells (*n* = 1-3) (B) with and without PrP^C expression were incubated with H_2O_2 for 24 hrs. The measured amount of oxidized bases (8-oxoG) assessed by LC-MS/MS is shown. Values are shown as mean ± SEM. (Significance tested by multiple t-test with Holm-Sidak correction, all p-values > 0.05).
