# Supplementary material for: Stress Resilience of Spermatozoa and Blood Mononuclear Cells without Prion Protein
Source: Front Mol Biosci. 2018 Jan 24;5:1. doi: 10.3389/fmolb.2018.00001 (PMC5787566; doi:10.3389/fmolb.2018.00001)
Supplement: Supplementary file 4 [file Image4.PDF]

Supplementary Material

Stress resilience of spermatozoa and blood mononuclear cells without prion protein

Malin R. Reiten[1], Giulia Malachin[1], Elisabeth Kommisrud[2], Gunn C. Østby[1], Karin E. Waterhouse[1,4], Anette K. Krogenæs[1], Anna Kusnierczyk[3], Magnar Bjørås[3], Clara M. O. Jalland[1], Liv Heidi Nekså[1], Susan S. Røed[1], Else-Berit Stenseth[2], Frøydis D. Myromslien[2], Teklu T. Zeremichael[2], Maren K. Bakkebø[1], Arild Espenes[1], Michael A. Tranulis[1*]

[1]Faculty of Veterinary Medicine and Biosciences, Norwegian University of Life Sciences, Oslo, Norway

2 Faculty of Education and Natural Sciences, Inland University of Applied Sciences, Hamar, Norway

[3]Department of Cancer Research and Molecular Medicine, Norwegian University of Science and Technology, Trondheim, Norway

[4]Spermvital AS Holsetgata 22, Hamar, Norway

*** Correspondence:**
Michael A. Tranulis
michael.tranulis@nmbu.no

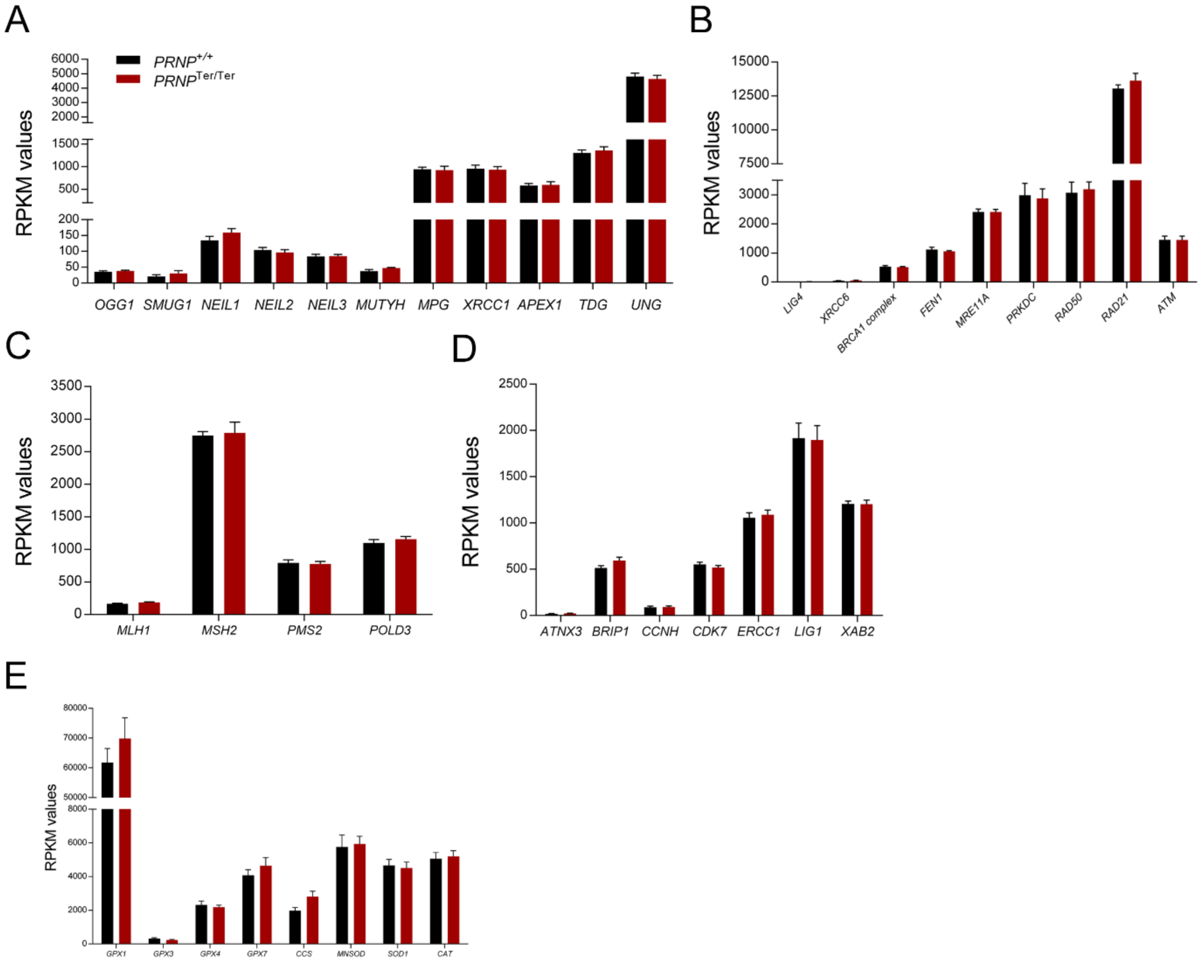

Supplementary Figure 4: Lack of prion protein does not affect expression levels of DNA damage-repair enzymes and antioxidant enzymes

RNA sequencing was conducted on peripheral blood mononuclear cells (PBMCs) with and without PrP^C expression. Transcriptomic analysis revealed no differences in the levels of major enzymes involved in the base excision repair (BER) (A), mismatch repair (MMR) (B), double strand break (DSB) (C) and nucleotide excision repair (NER) (D) pathways of DNA damage repair, and the levels of antioxidant enzymes (E) (n = 8, mean ± SEM, all $p > 0.05$). (Significance tested by multiple t-test with Holm-Sidak correction).
