# Supplementary material for: Stress Resilience of Spermatozoa and Blood Mononuclear Cells without Prion Protein
Source: Front Mol Biosci. 2018 Jan 24;5:1. doi: 10.3389/fmolb.2018.00001 (PMC5787566; doi:10.3389/fmolb.2018.00001)
Supplement: Supplementary file 5 [file Image5.PDF]

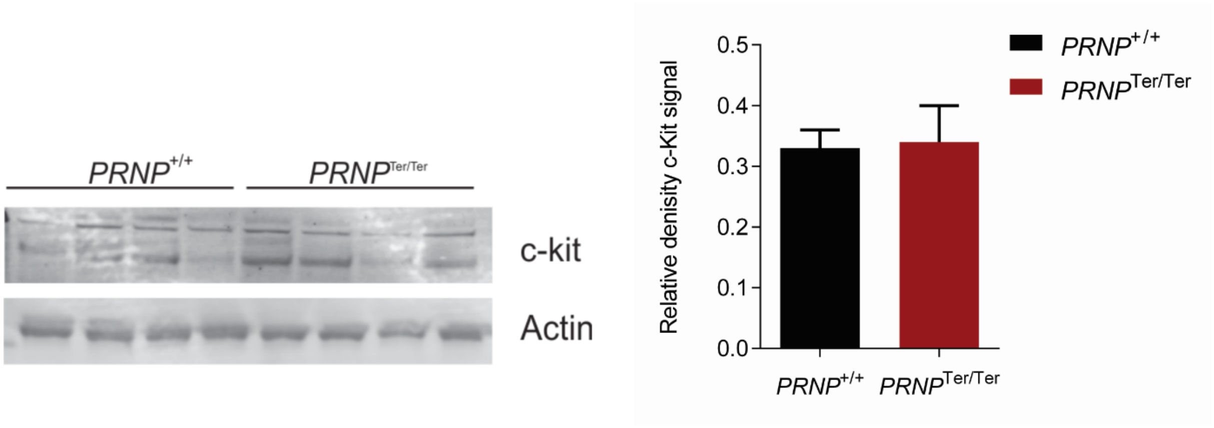

Supplementary Figure 5: Global testicular levels of c-Kit were similar between *PRNP*$^{+/+}$ and *PRNP*$^{Ter/Ter}$ bucks. Testicular homogenates from *PRNP*$^{+/+}$ (n = 4) and *PRNP*$^{Ter/Ter}$ (n = 4) were analyzed by western immune blot for semi-quantitative assessment of the stem-cell marker c-Kit. The data show individual variation but no major difference between the genotypes.
