# Supplementary figures and images for: Stress Resilience of Spermatozoa and Blood Mononuclear Cells without Prion Protein
Source: Front Mol Biosci. 2018 Jan 24;5:1. doi: 10.3389/fmolb.2018.00001 (PMC5787566; doi:10.3389/fmolb.2018.00001)

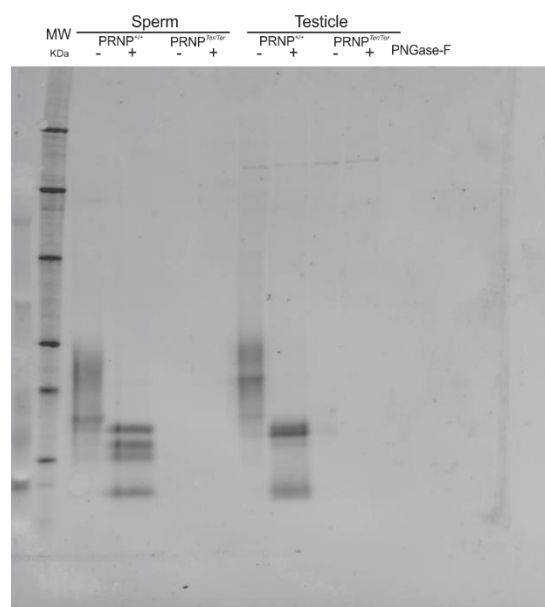

**Supplementary Figure 6: Uncropped image of Western blot membrane for Fig. 1A.**

Supplement: Supplementary file 6 [file Image6.PDF]

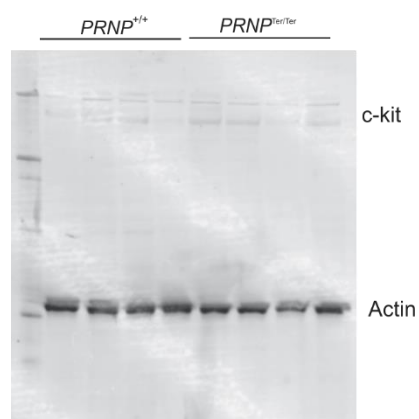

**Supplementary Figure 7: Uncropped image of Western blot membrane for Supplementary figure 5.**

Supplement: Supplementary file 7 [file Image7.PDF]
